# Widespread Site-Dependent Buffering of Human Regulatory Polymorphism

**DOI:** 10.1371/journal.pgen.1002599

**Published:** 2012-03-22

**Authors:** Matthew T. Maurano, Hao Wang, Tanya Kutyavin, John A. Stamatoyannopoulos

**Affiliations:** 1 Department of Genome Sciences, University of Washington, Seattle, Washington, United States of America; 2 Department of Medicine, University of Washington, Seattle, Washington, United States of America; Stanford University School of Medicine, United States of America

## Abstract

The average individual is expected to harbor thousands of variants within non-coding genomic regions involved in gene regulation. However, it is currently not possible to interpret reliably the functional consequences of genetic variation within any given transcription factor recognition sequence. To address this, we comprehensively analyzed heritable genome-wide binding patterns of a major sequence-specific regulator (CTCF) in relation to genetic variability in binding site sequences across a multi-generational pedigree. We localized and quantified CTCF occupancy by ChIP-seq in 12 related and unrelated individuals spanning three generations, followed by comprehensive targeted resequencing of the entire CTCF–binding landscape across all individuals. We identified hundreds of variants with reproducible quantitative effects on CTCF occupancy (both positive and negative). While these effects paralleled protein–DNA recognition energetics when averaged, they were extensively buffered by striking local context dependencies. In the significant majority of cases buffering was complete, resulting in silent variants spanning every position within the DNA recognition interface irrespective of level of binding energy or evolutionary constraint. The prevalence of complex partial or complete buffering effects severely constrained the ability to predict reliably the impact of variation within any given binding site instance. Surprisingly, 40% of variants that increased CTCF occupancy occurred at positions of human–chimp divergence, challenging the expectation that the vast majority of functional regulatory variants should be deleterious. Our results suggest that, even in the presence of “perfect” genetic information afforded by resequencing and parallel studies in multiple related individuals, genomic site-specific prediction of the consequences of individual variation in regulatory DNA will require systematic coupling with empirical functional genomic measurements.

## Author Summary

A comprehensive understanding of the contribution of individual genome sequences to disease and quantitative traits will require the general ability to predict consequences of genetic variation in non-protein-coding regions, particularly those involved in gene regulation. Here we tested the power to predict such consequences when presented with “complete” information encompassing the genomic DNA binding site patterns of a well-studied regulatory protein across multiple related individuals, coupled with all individual genome sequences at the binding positions. We find that, while there is reasonable ability to predict the average effects of variation within the consensus recognition sequence of a transcriptional regulator, it is not possible to determine reliably the consequences of variation at any given genomic instance. This suggests that the interpretation of individual genome sequences will require comprehensive complementation with functional genomic studies.

## Introduction

A growing number of studies associate variation within regulatory DNA and risk of human disease [1]–[3]. Variation in regulatory DNA may result in modulation of recognition by sequence-specific transcription factors (TFs), resulting in altered gene expression [4]–[6]. That the vast majority of variants emerging from human resequencing studies lie in non-coding regions creates an urgent need for determining the consequences of variation within regulatory DNA.

Functionally significant variation within the genomic recognition sequences for certain TFs appears to be correlated in aggregate with nucleotide-level evolutionary conservation and/or position-specific information content [7]–[10]. Although surveys have identified sites of allele-specific occupancy of TFs and RNA Polymerase II or allele-specific chromatin states [11]–[15], these studies have not established the distinguishing characteristics of regulatory sequence variation with an experimentally-observed effect on occupancy. As such, it is currently not possible to interpret reliably the functional consequences of variation within any given TF recognition sequence.

To address this, we apply a novel experimental design to identify comprehensively patterns of genetic variation with heritable effects on the occupancy of the major genomic regulator CTCF [16]. Unlike most sequence-specific regulators which rely on cooperative interactions with other factors to bind DNA, CTCF is able to access target DNA within chromatin in a relatively autonomous fashion through its rich binding interface. By combining quantitative genome-wide occupancy analysis by ChIP-seq in a multi-generational pedigree with comprehensive resequencing of the binding site landscape across all individuals, we achieve complete knowledge of variation in both sequence and occupancy, thus creating a benchmark for assessing the characteristics of functional and heritable regulatory sequence variation.

## Results

### Components of heritable transcription factor occupancy

**Figure 1:**
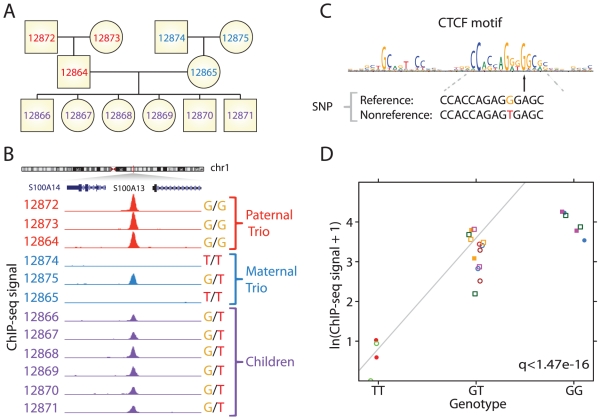
Systematic identification of the effect of genetic variation on transcription factor occupancy. (A) We performed ChIP-seq for the transcription factor CTCF followed by targeted resequencing of its complete occupancy landscape in 12 members of CEPH pedigree 1459 (CEU). (B) Three qualitative levels of occupancy correspond to three genotypes of a SNP located at the binding site, with G/G homozygotes having the highest occupancy (region shown: chr1:151,853,500–151,859,700 [hg18]). (C) The SNP shown in (B) disrupts a critical position in the CTCF consensus sequence (note that G better matches the consensus recognition sequence). (D) Regression of ChIP-seq signal on genotype at the site in (B) quantifies the effect of SNPs on occupancy. We applied this strategy genome-wide to identify sites where SNPs are associated with differences in occupancy. At this site, Akaike information criterion favored a dominant effect model (GT and GG coded identically) over an additive model.

We mapped binding sites for CTCF by ChIP-seq in B-lymphoblastoid cells derived from 12 members of a three-generation pedigree (Figure 1A, 1B). We identified a total of 51,686 binding sites across all individuals at a false discovery rate (FDR) of 1%. To comprehensively identify genetic variation with potential functional consequences for CTCF binding, we performed targeted resequencing by array capture focused on the 134 bp interval surrounding 46,568 CTCF sites (total ∼6 Mbp) in all family members assayed by ChIP-seq. 7,394 of the 35,709 surveyed binding sites (or 21%) overlapped one or more SNPs, some of which had clear associations with occupancy in the direction predicted by the CTCF motif (Figure 1C, 1D). We did not consider other variation such as copy number variants or small indels. In order to minimize reference mapping bias for the ChIP-seq data, we remapped tags to personalized genomes including discovered SNPs [17]. Additionally, to avoid artifacts resulting from uncertain mapping of 36 bp reads to the genome, we simulated all reads including discovered SNPs from ±147 bp centered on the ChIP-seq peak and excluded sites with too many ambiguously mapped tags.

**Figure 2:**
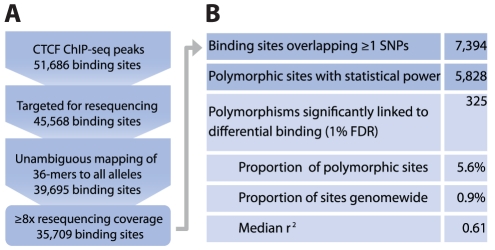
Genome-wide survey of the effect of genetic variation. (A) Filtering strategy for testable CTCF binding sites. A number of binding sites were excluded from the analysis due to microarray probe design constraints, poor mappability, differing mappability between two alleles, or insufficient resequencing coverage. (B) Summary of the prevalence of SNPs that affect CTCF occupancy at an FDR of 1%. Some sites overlapping SNPs were excluded for having insufficient data points per genotype to perform a robust regression. The model explained a substantial amount of the variance at significant sites (median r^2^ of 0.61).

We integrated the genetic and functional data sets to survey genome-wide heritable variation in transcription factor occupancy. We reasoned that the strongest signal of heritable variation would be from segregating variants overlapping the binding site. Thus we performed a linear regression of the ChIP-seq density on the SNP genotype in *cis* (Figure 1D). Of 5,828 polymorphic sites, this analysis identified 325 (5.6%) sites with a significant association of SNP genotype with occupancy at a false discovery rate (FDR) of 1% (Figure 2, Table S4). We tested whether several confounding factors might be responsible for our results, however sites at which SNPs were significantly associated with changes in occupancy were similar to polymorphic sites without changes in occupancy in terms of GC content (median 53.7% vs. 53.0%, Mann-Whitney p<0.044) and ChIP-seq input signal (3.61 vs. 3.61, Mann-Whitney p<0.84), and distance to the nearest RefSeq TSS (33 kb vs. 36 kb, p<0.96). Significant sites were only slightly weaker in terms of ChIP-seq density (2.73 vs. 2.93, p<2.7*10^−3^), and DNase I signal (5.47 vs. 6.92, p<7.8*10^−8^). Thus we conclude that the SNP genotype is associated with differences in occupancy at 325 of 5,828 sites tested.

We used a hypothesis-driven linkage analysis to assess the heritability of the remaining unexplained differential occupancy. First, we identified 1,376 sites of differential occupancy. Of these, 200 (15%) were already associated to an underlying SNP (FDR 1%), 65 (4.7%) had allele-specific occupancy (FDR 0.1%), and 197 (14%) were on chromosome X. To test for heritable inheritance of occupancy not explained by these factors, we performed Haseman-Elston sib-pair linkage analysis in aggregate at sites differentially occupied among the 6 grandchildren (Figure S1A and S1B; see Materials and Methods). The 47 binding sites already significantly associated with SNPs had a regression slope of −2.86 (Figure S1C), confirming substantial heritability (p<8.9*10^−7^, permutation). The remaining 50 sites without significant associations had a regression slope of −1.01 (Figure S1D), indicating a lower but still significant level of heritable variation (p<3.2*10^−5^). These results suggest that SNPs directly overlapping the cognate recognition sequence explain most but not all of the heritable variation in this pedigree. Remaining variation in occupancy might be heritable due to sequence variants not considered in our SNP-based analysis or heritable epigenetic variation such as methylation. However, it is quantitatively less significant than the heritability attributable to the direct effect of SNPs on occupancy (Figure S1E).

### Using motifs to align regulatory polymorphism from multiple sites

**Figure 3:**
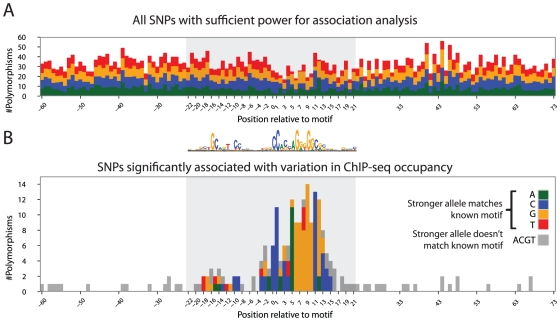
Functional SNPs recapitulate the CTCF binding motif. (A) 4,428 SNPs identified by resequencing at as many sites. Y-axis indicates the number of SNPs identified at a given position (x-axis) relative to the aligned and strand-oriented CTCF motif (below). Bar color indicates alleles of SNPs. Gray shading indicates the 44-bp extent of protein-DNA interaction. Note that SNPs are uniformly distributed throughout the entire window, except for a slight reduction in diversity corresponding to the high-information content positions of the motif. (B) Of the SNPs in (A), 218 are significantly associated with ChIP-seq occupancy (FDR 1%). Color indicates SNPs for which the higher-occupancy allele (according to association analysis) also had a higher log-odds score in the known motif. Gray indicates SNPs that affected occupancy, but the higher-occupancy allele had a lower score in the motif. See Figure S2C for full color. Note that these SNPs are concentrated in the region of protein-DNA contact, and 84% match the allele predicted by the canonical motif (above).

Understanding the effect of DNA sequence variation on transcription factor occupancy is critical to a mechanistic interpretation of non-coding variation. To interpret the association results in the context of the CTCF motif, we scanned the center of the ChIP-seq peak with the known position weight matrix (PWM) [18], which measures the contribution of each nucleotide in the binding site to the energy of the protein-DNA interaction [19], [20]. 888 binding sites did not contain a motif match (fimo p-value<10^−2^) and 1,040 binding sites overlapped multiple SNPs within ±180 bp of the ChIP-seq peak. Excluding these sites, we analyzed the 4,428 binding sites with a single SNP and a single motif match. These SNPs were distributed throughout the resequenced region surrounding each CTCF motif (Figure 3A).

In contrast, we expected that SNPs associated with occupancy differences would be concentrated in the 44 bp region of protein-DNA contact [21]. Indeed, despite a slight reduction (1.08-fold) in local sequence diversity, 85% of the SNPs that affected occupancy were within this region (Figure 3B). The allele observed to have higher occupancy matched the energetically more favorable one for 83% of these SNPs. Associated SNPs outside the region of contact had less significant q-values (median q-value of 1.3*10^−3^ outside the versus 1.3*10^−5^ inside), consistent with these SNPs being false positives or sites with ambiguity in the true location(s) of protein-DNA interaction. Alternatively, some of these SNPs might affect CTCF occupancy indirectly by perturbing an adjacent co-factor binding site.

We compared our results to an allele-specific occupancy test performed at heterozygous sites (Figure S2). Despite a weaker enrichment of significant sites within the core motif and a less substantial concordance of the higher occupancy allele with the energetically more favorable nucleotide, this allele-specific analysis broadly corresponded to the results of the association analysis. Thus, we interpret the results of our analysis as indicating that we have correctly identified the motifs at most binding sites, and that the significant SNPs directly affect occupancy through modulating the protein-DNA interaction at these sites.

### Context-dependent effect of identical changes

**Figure 4:**
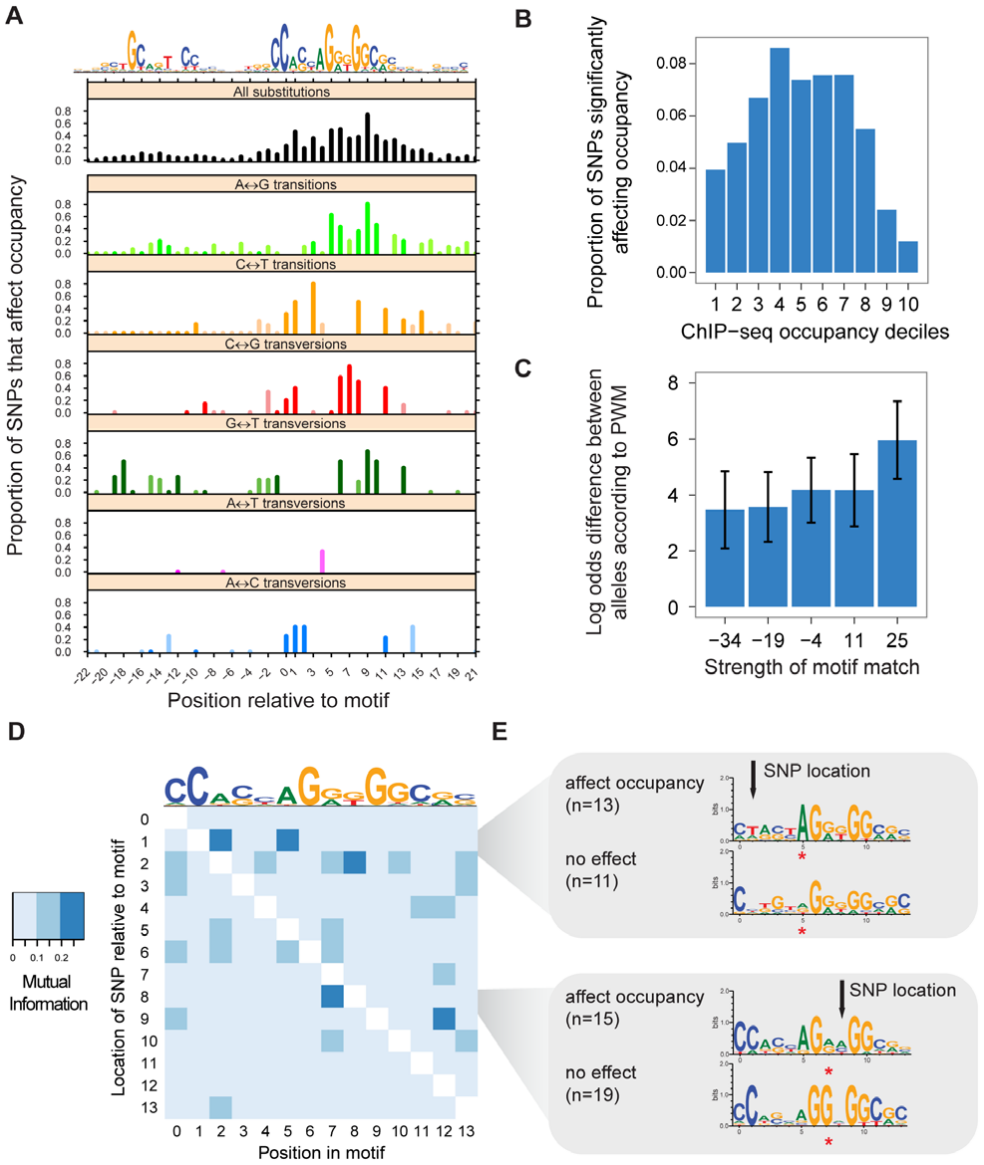
Sequence context buffers effect of polymorphism on occupancy. (A) Average effect of SNPs on occupancy across 1,368 different sites, broken down by genotypes (panels) and position (x-axis) relative to the canonical motif (top). Y-axis, proportion of sites where a change is associated with differences in occupancy (FDR 1%). In comparison, 1% of changes observed outside this 44 bp region affected binding (Table S4). Only changes observed at least 3 sites are considered; in particular, few A–T transversions were observed due to the GC-rich nature of the motif. (B) SNPs at the weakest and strongest sites are less likely to affect occupancy. X-axis, decile of ChIP-seq signal for the heterozygote genotype according to the regression model; each decile represents 583 sites. Y-axis, proportion of sites in at which SNPs are associated with differential occupancy. (C) SNPs affecting occupancy despite stronger motif contexts involve more severe perturbations. X-axis, log-odds score of motif match, stronger matches at the right, label represents lower limit of bin. Y-axis, magnitude of perturbation, represented by the difference in log-odds scores between the two alleles. Error bars indicate standard deviation. In contrast, SNPs not affecting occupancy show no such trend (Table S9). (D) Each cell measures the mutual information between the base pair at positions in the core motif (x-axis) and whether a SNP at another position in the motif (y-axis) affects occupancy (FDR 5%). (E) Sequence context at sites with SNPs (arrows) at position 1 (above), 6 (below), divided by whether the SNP affected occupancy. Red stars highlight significant sequence differences (q<0.05, see Materials and Methods) between buffered and unbuffered sites at positions with elevated mutual information along the x-axis in (D).

Although differences in occupancy were largely associated with SNPs at positions strongly affecting overall binding energy (Figure 3B), only 13% of SNPs at the interface of protein-DNA interaction affected occupancy (Table S4). Thus although functional SNPs are highly concentrated in the region of protein-DNA contact, the majority of SNPs, even in this region, do not measurably affect occupancy. Since our data set includes multiple sites with the same two alleles at the same position measured relative to the binding motif, we investigated the proportion of sites at which a given change was found to affect occupancy (Figure 4A). Like in Figure 3, the most disruptive changes were observed at positions of high information content in the motif. However, even over the 14 bp core motif, changes affected occupancy at a median of only 36% of the sites where they were observed. We found no changes that uniformly affected occupancy without regard to context. Instead, we observed a strong, progressive depletion in the proportion of changes affecting occupancy at the strongest sites (Figure 4B), and a smaller depletion at the weakest sites. Indeed, simply clustering the ChIP-seq intensities identified three major groups, of low, medium and high occupancy, which were also distinguished by varying proportions of significant SNPs (Figure S6). This result places an upper limit on the accuracy of methods that predict the effects of non-coding SNPs without consideration of their context.

Strength-dependent buffering could be explained by a model where changes in occupancy are observed only when a SNP causes the affinity of a site to cross a threshold for binding. In this case, the strongest and weakest sites will only be affected by the greatest genetic perturbation, while smaller perturbations would affect binding only at sites of intermediate strength. This would create the impression of epistasis between all positions in the cognate recognition site as any affinity-affecting change could potentially buffer another [22], [23]. We thus compared the inherent affinity of the site with the magnitude of the perturbation caused by each SNP. We divided SNPs affecting occupancy into bins based on the strength of their match to the canonical motif. We found that SNPs at sites matching the CTCF motif more strongly in turn exhibited higher log-odds differences (Figure 4C). We observed no such trend at SNPs not associated with occupancy differences (Figure S3). These results are consistent with stronger motifs being buffered against all but the largest perturbations. Although a linear regression identifies a significant effect (p<0.006), an r^2^ of 0.04 indicates that the strength of the motif match alone can not explain the breadth of buffering observed.

Buffering might also be a consequence of the non-additive effect on binding energy of individual positions in the cognate binding site, as has been observed *in vitro* [24]. The relevance of non-additive interactions for identifying binding sites has been questioned [25], [26], but the implications for understanding the function of specific variants *in vivo* have remained unclear. To explore the power of our data set to discover epistatic interactions, we measured the mutual information between the sequence context per-base in the core motif and whether a SNP at each location affects occupancy (Figure 4D). This analysis identifies two positions in the consensus sequence that significantly buffer the effect of a SNP at another position. First, of the 24 SNPs observed at position 1 in the motif, 13/13 that affected occupancy had an adenine at position 5, compared to only 5/11 for those that did not affect occupancy (Figure 4E, above). Interestingly, the second significant buffering interaction is between position 7 and SNPs at the adjacent position 8 (Figure 4E, below), suggesting local compensation for the adjacent SNP. These results indicate that higher-order models may be necessary to fully model the effect of polymorphism on protein-DNA interaction, and are consistent with a model where local factors determine whether polymorphism affects occupancy.

### Power to predict functional polymorphism in non-coding regions

**Figure 5:**
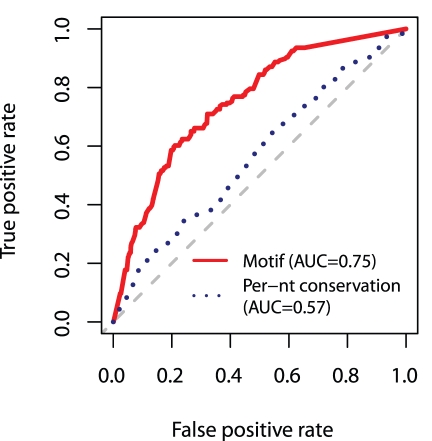
Power of existing measures to predict the effect of regulatory polymorphism. ROC curve evaluating the power of two measures on the 1,368 SNPs in this study found within the region of protein-DNA contact, 186 of which significantly affect occupancy. Dotted blue line represents predictions by ranking SNPs in decreasing order of inferred purifying selection (phyloP per-nucleotide conservation score) at the location of the SNP. Solid red line represents predictions by ranking SNPs based on the difference in log-odds scores between alleles. Area under the curve (AUC) summarizes overall predictive power. Gray line indicates a random predictor and has an AUC of 50%. A perfect predictor would be plotted as a right angle, ranking all functional SNPs ahead of all nonfunctional SNPs, and would have an AUC of 1.0. While per-nt conservation performs little better than chance, consideration of binding energetics substantially improves performance.

Resequencing and association studies are producing large amounts of data on polymorphism in non-coding regions, yet as we have illustrated, their functional classification is difficult. To investigate the power of existing metrics to predict functional polymorphism in non-coding regions, we used as a reference set the 1,368 sites with SNPs within the 44 bp vicinity of a recognizable CTCF motif. We first assessed the predictive power of evolutionary constraint, which has been used successfully to discover regulatory motifs [27], to highlight functional positions within motifs [9], [10], [28], and to predict the effect of coding variants [29]–[31]. Conservation is a particularly attractive operational metric in genome scans, as it can be applied in an unbiased fashion without directly measuring protein-DNA interaction or modeling context effects. Indeed, CTCF binding sites are clearly marked by increased conservation [32]. Thus, we tested the sensitivity and specificity of per-nucleotide conservation (phyloP 44-way vertebrate alignment, from UCSC browser) to correctly identify the 186 significant SNPs in our reference set. However, despite being applied only across experimentally determined binding sites, conservation had little predictive power on this data set, with an AUC of 0.57 (Figure 5).

Then we measured the improvement from evaluating potentially functional SNPs within the context of protein-DNA binding energetics. Applying such predictor showed a marked improvement over conservation, with an AUC of 0.75 (Figure 5), although positive predictive power was greatest for the most severe perturbations (Figure S4). Nevertheless, these results illustrate the power to be gained from considering non-coding polymorphism within the context of functional genomics data on transcription factor occupancy.

## Discussion

In contrast to the vast diversity of protein function, the elements that regulate gene expression recruit from a shared repertoire of transcription factors, offering the potential for a common regulatory sequence code. The torrent of variants emerging from human resequencing studies – the vast majority of which lie in non-coding regions – coupled with the growing number of common, disease-associated non-coding variants [1]–[3] has created an urgent need for determining the consequences of variation within regulatory DNA. However, the proportion of variants within regulatory DNA that have reproducible functional consequences on regulatory factor binding is currently unknown, and our ability to predict such outcomes from known rules of protein-DNA interaction is uncertain.

We have described a novel, hypothesis-driven genetic method employing targeted capture and genome-wide *in vivo* occupancy profiling to investigate directly the consequences of heritable variation in regulatory sequence. Our results show that individual transcription factor binding sites are surprisingly robust to genetic variation, even at evolutionarily constrained positions. While previous studies have observed differences in transcription factor occupancy among individuals using occupancy profiling alone [15], genome-wide linkage scans [33], or allele-specific occupancy approaches [11], this work is the first systematic analysis of patterns of functional alteration in TF recognition sequences. This study further advances the characterization of heritable variation in TF binding by using highly accurate sequence information throughout a three-generation pedigree.

Our study has revealed a large degree of context dependence for changes to the CTCF recognition sequence. Indeed, even over the core 14 bp motif, only 36% of SNPs affected occupancy (Figure 4A). Our estimate of the percentage of SNPs that affect occupancy in this 14 bp region ranges between 24% to 42% at FDRs 0.1% and 5%, respectively, indicating that the magnitude of this effect cannot be explained by the choice of significance cutoff. We have suggested that buffering is partly mediated by the strength of the binding site, as well as the sequence context at the local CTCF recognition sequence. In addition, buffering might be facilitated by a feedback process that maintains a constant CTCF occupancy despite alterations to the site's inherent affinity. However, while 21% of the SNPs in the region of protein-DNA contact that were significant in our association analysis also exhibited allele-specific occupancy in heterozygous samples, only 3.7% of the non-significant SNPs did, indicating that buffering is not likely to be the consequence of a feedback process. Alterations in DNA methylation might also mask the effect of otherwise significant genetic changes. However, only 30% of polymorphic CTCF sites contain a CpG at positions 1 or 11 of their recognition sequences. Furthermore, the prevalence of CpGs at these positions is the same at sites where a SNP does and does not affect occupancy, limiting the potential scope of methylation to fully explain the observed buffering. As this study was performed on transformed B-lymphoblastoid cells, it is worth noting that the specific CTCF sites that are buffered may not be extrapolated to primary cell types. However, assuming that EBV transformation does not invoke novel cellular mechanisms to regulate protein-DNA interaction, our primary conclusion stands that TF occupancy is strongly modulated by site-dependent effects.

Our results establish a low level of mutational load directly affecting transcription factor occupancy in the 4 founder genomes. Although variants were found at 21% of surveyed CTCF sites, only 0.9% of binding sites exhibited a difference in occupancy due to polymorphism (Figure 2). Previous studies have identified varying levels of positive and negative selection in transcription factor recognition sequences by estimating changes in binding energy [34]–[36]. However, our results indicate that 87% of polymorphism observed in the region of protein-DNA contact does not affect binding (Table S4). This is a higher proportion of silent variation than predicted by binding energy models (Figure S4), providing evidence that the scope of sequence change consistent with neutral evolution may be larger than previously thought.

Interestingly, we observed that the allele with higher occupancy was the derived allele in 40% of the cases (assuming the chimpanzee allele is ancestral). This indicates that approximately 40% of the functional substitutions in the human lineage increased occupancy, which is surprisingly high given that most mutations might be expected to reduce binding energy.

Previous work studying the power of comparative genomics has predicted a steep increase in the number of sequenced genomes required to obtain nucleotide resolution, particularly in the absence of perfect conservation [37]. While genome-wide phylogenetic footprinting approaches have highlighted substantial conservation of transcription factor sequence specificities [27], [32], [38], [39], functional studies of diverged species have uncovered low conservation in occupancy at orthologous sites [40]–[42]. Any phylogenetic approach is thus a compromise between statistical power gained by sampling more diverged species and the ability to recognize similar functional elements by sequence similarity. The optimum evolutionary distances to sample may be different for assessing functional non-coding elements than for more conserved coding sequence [43], [44]. This tradeoff suggests a potential motivation for broad resequencing of natural populations, though even this approach faces the fundamental limitation of ineffective purifying selection in primates and humans [45].

Gene-based studies have successfully identified causal variants using current methods for prediction of functional non-synonymous protein variants [46]. Coding mutations in the CTCF gene affecting its DNA binding specificity have been identified in cancer samples [47], but lesions in its binding sites are harder to interpret. The link between cognate recognition sequence and cellular consequence is complicated by potential influences from the cell-type specific chromatin landscape [48], maintenance of regulatory function despite sequence rearrangement [49], altered association with protein complexes [50], [51], and the lack of binding specificity and occupancy data for common transcription factors. In spite of these caveats, our results indicate that a motif-based classifier of variation in experimentally-identified CTCF binding sites predicts functional variation with a 59% true positive rate and a false positive rate of 20% (Figure 5). In comparison, current methods for prediction of non-synonymous protein variants achieve a 73% to 92% true positive rate at the same false positive rate [30] – not dramatically greater considering the comparatively greater depth of variant databases used to assess coding variation. Given the encouraging performance of a straightforward functional genomics approach, that the majority of variants presently associated with human physiology and pathology lie in non-coding regions [3] should be grounds for optimism.

In summary, our results indicate the existence of widespread recognition site-dependent buffering of polymorphism within regulatory DNA regions. A major implication of our work is that the potential for accurately predicting the consequences of variation affecting regulatory factor recognition sequences is severely limited by complex context dependencies, necessitating empirical assessment using functional genomic approaches. The feasibility of approaches such as the one we describe here has recently dramatically increased owing to coupled advances in next-generation sequencing technology and molecular biology, and continuation of this trend should in the near future enable further systematic investigations into the effect of polymorphism on protein-DNA interaction on a routine basis.

## Materials and Methods

### Cell culture

The B-lymphoblastoid cell lines from CEPH pedigree 1459 were obtained from Coriell and cultured in RPMI1640 medium (Cellgro), supplemented with 15% fetal bovine serum (FBS, Hyclone), 2 mM L-Glutamine, and 25 IU/mL penicillin and 25 µg/mL streptomycin (Cellgro).

### ChIP–seq

B-lymphoblasts (5 million cells) were crosslinked with 1% formaldehyde (Sigma), lysed in lysis buffer (50 mM Tris-HCl pH 8.0, 10 mM EDTA pH 8.0, 1% SDS), and sheared by Bioruptor (Diagenode). The supernatant was further diluted 10-fold with dilution buffer (50 mM Tris-HCl pH 8.0, 166 mM NaCl, 1.1% Triton X-100, 0.11% sodium deoxycholate). For each immunoprecipitation, 100 µL Dynabeads (M-280, sheep anti-rabbit IgG, Invitrogen) were incubated with 20 µL CTCF antibody (#2899, Cell Signaling) for at least 6 hours at 4°C. The antibody-conjugated beads were then incubated overnight with sheared chromatin. The complexes were washed with IP wash buffer I (50 mM Tris-HCl pH 8.0, 0.15 M NaCl, 1 mM EDTA pH 8.0, 0.1% SDS, 1% Triton X-100, 0.1% sodium deoxycholate), high salt buffer (50 mM Tris-HCl pH 8.0, 0.5 M NaCl, 1 mM EDTA pH 8.0, 0.1% SDS, 1% Triton X-100, 0.1% sodium deoxycholate), and TE buffer (10 mM Tris-HCl pH 8.0, 0.1 mM EDTA pH 8.0). Crosslinking was then reversed in elution buffer (10 mM Tris-HCl pH 8.0, 0.3 M NaCl, 5 mM EDTA pH 8.0, 0.5% SDS) at 65°C overnight. The DNA was separated from the beads and treated with Proteinase K (Fermentas) and purified by phenol-chloroform extraction and ethanol precipitation.

Sequencing libraries were constructed according to Illumina's genomic prep kit protocol as previously described [28]. Briefly, ChIP DNA was end-repaired using the End-it DNA repair kit (Epicentre). Adenines were added to the 3′ ends of the blunt-ended DNA using Taq DNA polymerase (NEB). PE adapter (1∶20 dilution, 1 µL for 15–50 ng starting ChIP DNA, Illumina) was ligated to the ends of the A-tailed ChIP DNA with T4 DNA ligase (NEB). 1/3-1/4 of the purified ligation product was PCR amplified for 16 cycles with High-fidelity PCR master mix (NEB) and PE primer 1.0/2.0 (Illumina). Libraries were run on 2% agarose gels, size-selected, and purified with QIAquick gel extraction kit (Qiagen). The libraries were sequenced to 36 bp on an Illumina Genome Analyzer by the High-throughput Genomics Unit (University of Washington) according to standard protocol. ChIP-seq experiments were performed on 2–3 independently cultured biological replicates per sample (Table S1, Table S2).

### ChIP–seq peak calls

High quality reads were aligned to the reference genome using the Eland aligner. SPP [52] was used to call peaks on total tag data from the 12 samples, resulting in 51,686 peaks at a Poisson-derived FDR of 1%. Using the aggregate of all tags was more conservative (in terms of number of peaks) than taking the union of peak calls on individual samples, but less conservative than taking the intersection. The MTC method (“tag.lwcc”) was used to call point binding positions (Table S5). Motif representations used Weblogo [53].

### Capture resequencing

We designed an Agilent 244k SureSelect microarray for targeted resequencing on the 51,686 ChIP-seq peaks identified in the 12 samples. We used fimo (http://meme.sdsc.edu/meme/) to scan for instances of the core 14 bp of the canonical motif [18] with a p-value of 10^−2^. We adjusted the target locations to center on matches to the nearest CTCF motif if the motif was within 50 bp, and added flanking targets to capture additional nearby motifs. 5 potential probes were tiled at 15 bp spacing to the 120 bp surrounding each target. We adjusted probe binding energy similarly to Ng et al. [54], adjusting the spacing of probes by up to 5 bp and adjusting the lengths to between 40–60 bp to reach a predicted T_m_ between 60–72°C. We used the Duke Uniqueness 20 bp track (UCSC genome browser) to filter out 5,828 probes with potential for cross-hybridization. We further excluded 145 probes in satellite repeats (RepeatMasker, UCSC genome browser) or with high blast scores to multiple genomic locations. The final design had 242,380 probes targeting 46,652 CTCF sites.

Genomic DNA was extracted from cultured cells, and targeted capture was performed based on Supplementary Protocol 3 of Mamanova et al. 2010 [55] with modifications. Briefly, 12 µg of genomic DNA was sheared in a Covaris S2 (Covaris, Inc.) using a duty cycle of 10%, intensity 5, cycle/burst 100, time 600 sec. DNA was end-repaired, A-tailed, and ligated to SE adapters (Illumina), and purified with Agencourt AMPure XP beads (Beckman Coulter). A trial PCR amplification using Phusion HF polymerase (NEB) and SE primers SLXA_FOR_AMP and SLXA_REV_AMP [54] (IDT) was performed on a fraction of ligated DNA with an Roche LightCycler 480 to determine the optimal number of cycles. PCR for all ligated DNA was then performed in eight 200 µL tubes using the following program: 98°C for 30 s, then a previously determined number of cycles of 98°C for 10 s, 65°C for 30 s, 72°C for 30 s, followed by 72°C for 5 min. The final DNA library was pooled and concentrated to 5 µL in a SpeedVac. For hybridization, 10 µg of DNA library was combined with formamide (Ambion), blocking oligos (SLXA_FOR_AMP, SLXA_REV_AMP, SLXA_REV_AMP_rev and SLXA_FOR_AMP_rev [54], Human C_0_t-1 DNA (Invitrogen), 2× Hi-RPM Hybridization Buffer (Agilent) and 10× Blocking Buffer (Agilent). Hybridization was performed using the Maui Hybridization System (BioMicro Systems, Inc.) at 55°C for 48 hours according to “MAUI Mixer LC on Agilent 244K CGH Microarrays” protocol. After hybridization, microarrays were washed with Agilent aCGH Wash Buffers 1 and 2, sealed with Secure-Seal (GRACE Bio-Labs) and placed on heat block (VWR) for elution of DNA. DNA was eluted with 3 mL of 95°C PCR-grade water, concentrated, and amplified with SE primers using the same PCR program as above. The libraries were sequenced to 36 bp on an Illumina Genome Analyzer by the High-throughput Genomics Unit (University of Washington) according to standard protocol.

Reads were aligned to the human genome (hg18) using bwa 0.5.8 [56] using default settings, allowing up to 2 mismatches (Table S3). Some lanes exhibited an excess of mismatches to the reference sequence at the 5′ or 3′ end, so tags were trimmed by up to 9 bp. Reads with identical 5′ ends were presumed to be PCR duplicates and were excluded using Picard v1.22 MarkDuplicates (http://picard.sourceforge.net/). SAMtools v0.1.7 [57] was used to generate a pileup of potential SNPs from uniquely-mapping reads with a mapping quality above 30. SNPs were called (Table S6) as biallelic variants with at least 8× resequencing coverage, a Phred-scaled SNP quality of at least 30, at least 20% of reads matching the allele with lower coverage, and Mendelian segregation according to PLINK v1.07 [58]. The chimpanzee allele was identified using axtNet alignment files for PanTro2 from the UCSC Genome Browser.

We performed two validations of the SNP calls from our targeted resequencing. First, we performed Sanger sequencing on PCR products from genomic DNA (Table S8). We tested 33 SNPs in all 12 samples. 0 of the 388 genotype calls were discordant. Second, we compared genotypes with genotypes available from the HapMap project for 6 of the 12 samples (Table S9). Release 27 genotypes were obtained from http://hapmap.ncbi.nlm.nih.gov, and matched to capture resequencing SNPs by location. 244 of the 27,808 genotype calls in common (0.88%) were discordant.

### Measuring occupancy differences among individuals at a common set of peaks

For each individual, a custom human genome was created from hg18 including autosomes, unscaffolded contigs, mitochondrial DNA, a Y chromosome for males, and the Epstein-Barr virus genome (gi|9625578|ref|NC_001345.1). Each genome was personalized to reflect SNPs identified by resequencing, using IUPAC codes to represent heterozygous position. ChIP-seq data was mapped using MosaikAligner v1.1.0021 (http://code.google.com/p/mosaik-aligner/) with the options “-bw 13 -act 20 -mhp 100 -m unique -mm 4 -minp 1.0”, requiring a unique mapping considering up to 4 mismatches. Reads with more than 2 mismatches were then discarded. Reads with identical 5′ ends were presumed to be PCR duplicates and were excluded using Picard v1.22 MarkDuplicates. Smoothed density tracks were generated using bedmap (http://code.google.com/p/bedops/) to count the number of tags overlapping a sliding 150 bp window, with a step width of 20 bp. Density tracks were normalized for sequencing depth by a global linear scaling to fix an arbitrary value of 25 as the 50th percentile of bins with more than 15 reads. We identified instances of the canonical motif (fimo p-value<10^−2^) within 15 bp of the center of the resequencing target, keeping the motif with the best p-value. We measured occupancy by the maximum normalized ChIP-seq tag density over the 14 bp motif.

### Regression of occupancy on genotype

We applied a regression method to measure whether a particular biallelic SNP is associated with occupancy, and if so which allele is associated with higher occupancy. We tested only sites with ≥8× resequencing coverage in ≥6 samples, sufficient mappability, and data for ≥4 data points each for ≥2 genotypes and ≥12 data points overall (Table S7). We further excluded 242 sites overlapping indels in the CEU population identified by the 1000 Genomes Project release 2010_07 [59]. We used a negative binomial generalized linear model (glm.nb in the R package MASS) to measure the significance of the effect of polymorphism on occupancy using an additive effect model, and including a replicate term to account for batch effects. We used two ChIP-seq replicates per sample, except for GM12870, which had one replicate (Table S1).

For sites with more than one SNP, we tested only the SNPs with more data points and those inside the region of protein-DNA contact. If there were still multiple SNPs per window, we chose the SNP with the lowest p-value, though these sites were then excluded from analyses depending on the known position of the SNP relative to the motif. We also tried fitting a dominant effect model where permitted by sample size: we chose between additive and dominant effect models using the Akaike information criterion. We separately fitted a linear model on the same data to estimate the r^2^.

We used the R package qvalue to estimate q-values (Figure S5) [60], which established a cutoff of p<9.6*10^−4^ as an FDR of 1% (325 sites). Using a Benjamini-Hochberg FDR strategy confirms a similar cutoff for FDR 1% (4.9*10^−4^, 293 sites). To independently confirm our FDR methodology, we considered the proportion of significant SNPs within the region of protein-DNA contact (Figure 3), which ranged from 71%, to 85%, to 91% at FDRs 5%, 1%, 0.1%, respectively (Table S4).

### Aggregate Haseman-Elston linkage analysis

We used 31,128 SNPs identified in our resequencing as markers to generate a map of identity-by-descent (95^th^ percentile marker spacing of 0.5 cM). We used recombination rate data [59] to place our SNP coordinates onto a genetic map, choosing SNPs with fewer missing genotypes at duplicate map positions. We then used MERLIN [61] to filter out improbable genotypes and compute IBD at our marker locations (option “–ibd”). We used the nearest marker at each binding site to estimate IBD for the 15 possible sib pairs. IBD values were placed into 3 bins (0, 0.5, and 1.0); values with >0.05 uncertainty were excluded.

We then used the package DESeq [62] to identify differentially occupied regions, both throughout all 12 samples as well as among just the grandchildren, using two replicates per sample (Table S1). We then applied a variance stabilizing transformation, and normalized the occupancy at each site to a mean of 0 and standard deviation of 1. We then averaged the occupancy of the two replicates.

We then performed Haseman-Elston regression at the 97 autosomal sites differentially occupied among the grandchildren, treating separately sites whose differential occupancy was already associated with a SNP or allele-specific binding. We considered all sib pairs and all sites simultaneously. Although regression methods exist with higher power [63] or that use data from all members of the pedigree [64], we applied the original method of regressing squares of trait differences for sib pairs, reasoning that the robustness of a simple method would have more forgiving assumptions. To account for the non-independence of measuring multiple sib pairs from the same family, we assessed the significance of the regression slope by permuting IBD vectors to random sib pair difference vectors for all differentially occupied sites, thus maintaining any correlation structure in the data.

### Allele-specific occupancy analysis

We tested allele-specific occupancy in pooled replicate data for each sample. Given the reliance of allele-specific occupancy tests on high coverage at heterozygous sites, we included data from an additional replicate for 4 samples (Table S1). We had sufficient power to test 2,535 heterozygous binding sites with adequate mappability and 13× ChIP-seq read coverage. We used a chi-squared test against a 50∶50 null expectation to derive a p-value from the counts of the ChIP-seq tags mapping to the two alleles. We used the R package qvalue to estimate an FDR (Figure S5) [60]. In interpreting our FDR threshold, we considered the observed concentration of significant SNPs within the 44 bp region of protein-DNA interaction (Figure S2), which increased at more conservative FDRs: 63%, 66%, 68% at FDRs 1%, 0.5%, 0.1%, respectively (Table S4). At sites for which multiple samples had testable heterozygous sites with the same alleles, the sample with the most total reads was picked as representative for plotting.

### Position weight matrix (PWM) motif models

We used PWM models [19] to measure the effect of a polymorphism on information content. CTCF binds in a multivalent fashion [21], [65], [66], wherein three modes of binding are distinguished by the presence and position of an upstream motif. At each site we chose the best-matching of the three motif models. To measure information content, we converted frequencies to log-odds, using a pseudo-frequency of 0.01 (the minimum observed frequency).

### Mutual information analysis

 We calculated the mutual information between whether a given SNP affected occupancy (FDR 5%) and sequence context at 14 positions in the core CTCF motif using the mutualInfo function of the R package bioDist. To estimate the significance of the mutual information values, we applied a bootstrap method for each pair of positions tested. We calculated p-values from 2,000 iterations of resampling per pair of positions (Figure S7). To account for multiple testing across all positions, we used the R package qvalue [60].

### ROC curve

We downloaded phyloP based on a 44-way vertebrate alignment from the UCSC Genome Browser. SNPs were ranked in decreasing order of the phyloP score at the location of the SNP, thus ranking sites with the most indication of purifying selection the likely to be disrupted by a SNP. To measure the predictive power of models of protein-DNA binding energy, we used a position weight matrix to compute the difference in log-odds score between the two alleles of each SNP. Sites with the largest difference between alleles at the location of the SNP were ranked as most likely to be disruptive. Plots were generated using the R package ROCR [67].

### Data availability

ChIP-seq data are viewable in the UCSC Genome Browser (http://genome.ucsc.edu, version hg18), and have been deposited in GEO (GSE30263). Resequencing data are available in the SRA (SRP009457), and the capture resequencing array design in GEO (GPL14147).

## Supporting Information

Figure S1 Aggregate linkage analysis confirms that association analysis identifies most but not all heritable signal. (A) Example site showing differential occupancy among the 6 grandchildren (B) The alleles transmitted to each grandchild illustrate identity by descent (IBD). PF, paternal grandfather; PM, paternal grandmother; MF, maternal grandfather; MM, maternal grandmother. Note that the grandchildren who share alleles from the same grandparent have more similar occupancy; compare the ChIP-seq signal in (A) for the grandchildren who inherited an allele from the paternal grandmother (PM) for (GM12867, GM12868 and GM12870) to the signal for those who inherited an allele from the paternal grandfather (PF; GM12866, GM12869 and GM12871). (C, D) Aggregate Haseman-Elston linkage analysis sites exhibiting differential occupancy in the 6 grandchildren, analyzing occupancy at (C) a positive control of 47 autosomal sites where already associated with a SNP or (D) the remaining 50 autosomal sites not significantly associated with a SNP. A negative slope indicates that sib pairs with more similar inheritance at the binding site also exhibit more similar occupancy at that site, thus implying that the variation in occupancy is heritable. (E) Permutation analysis to quantify significance of slopes from (C, D). Both sets of sites (arrows) show significant heritability compared to permuted data, and the signal is weaker for sites not directly implicated in the association analysis (right arrow).
(EPS)

Figure S2 Comparison of association and allele-specific analyses. (A–B) Y-axis indicates the number of SNPs tested for (A) association with occupancy and (B) allele-specific occupancy at a given position (x-axis) relative to the known CTCF motif (below). Color indicates alleles of the SNPs at each position. Gray shading indicates the extent of protein-DNA contact. Although SNPs were found distributed uniformly throughout the resequencing window, note that in (B) fewer SNPs are testable overall and at the flanks given the need for heterozygous SNPs with high ChIP-seq coverage, despite several additional replicates. We observed a slight reduction in SNPs directly over the core motif, corresponding to the most evolutionarily conserved region. (C–D) SNPs significantly associated with differences in ChIP-seq occupancy (C) and sites demonstrating allele-specific occupancy (D). Color indicates higher-occupancy allele. Both analyses demonstrate an enrichment of significant SNPs over the region of protein-DNA contact, though to a greater extent in (C). (E–F) Same as (C–D), but color indicates SNPs for which the higher-occupancy allele (according to association analysis) also has a higher log-odds score in the canonical motif and gray indicates SNPs that affect occupancy, but the higher-occupancy allele has a lower score in the motif. In comparison to the allele specific occupancy (F), the regression analysis (E) identifies more sites (325 vs. 181), has a higher concentration of significant SNPs within the region of protein-DNA contact (85% vs. 66%), and has a higher proportion of SNPs matching the expected binding energetics (84% vs. 68%). (A, E) are reproduced from Figure 3.
(EPS)

Figure S3 SNPs not affecting occupancy show no relationship between the magnitude of the perturbation and the strength of motif match. Y-axis, magnitude of perturbation, represented by the difference in log-odds scores between the two alleles. X-axis, log-odds score of motif match, stronger matches to the right, label represents lower limit of bin.
(EPS)

Figure S4 Positive predictive value of PWM model to predict the effect of SNPs on occupancy. X-axis measures stringency of cutoff, represented by the difference in log-odds scores between alleles. At a log-odds difference cutoff of 7.1, 41% of predictions represent true positives.
(EPS)

Figure S5 Statistical identification of association with differential occupancy and allele-specific occupancy. (A–C) Association of SNPs in *cis* with ChIP-seq occupancy (A) Histogram of p-values for all tested binding sites (B) Histogram of FDR-adjusted q-values (C) Effect size of SNPs associated with differences in occupancy (FDR 1%), measured by r^2^ of a linear regression (D–F) Allele-specific occupancy at heterozygous sites (D) Histogram of p-values for all tested binding sites (E) Histogram of FDR-adjusted q-values (F) Effect size of SNPs associated with allele-specific occupancy (FDR 0.005%), measured by the log of the ratio of the counts of reads mapping to the higher and lower alleles.
(EPS)

Figure S6 Hierarchical clustering confirms strength-dependent buffering. (A) Normalized ChIP-seq density for 379 CTCF sites (Y-axis) with polymorphism in the core region of protein-DNA contact (positions 0–13) in the six grandchildren (X-axis). Hierarchical clustering resolves three clusters (labeled at right). (B) However, the percentage of polymorphisms that significantly affect binding is higher in cluster 3 (42%) than in cluster 1 and 2 (33% and 27%). (C) The sites comprising the three clusters are distinguished by their overall ChIP-seq intensity, with cluster 1 being the weakest sites, cluster 3 being intermediate, and cluster 2 being the strongest sites; compare to Figure 4B.
(EPS)

Figure S7 Statistical significance of the mutual information between sequence context and SNPs affecting occupancy. P-values estimated by bootstrap; two interactions with p<0.0025 were considered significant (q<0.05, see Materials and Methods).
(EPS)

Table S1 Summary of ChIP-seq data mapped to customized genomes with Mosaik. Tags used in analysis indicates the uniquely aligned tags remaining after removing duplicates. Enrichment indicates the proportion of tags mapping to a ChIP-seq peak, representing the enrichment over background and indicating the quality of the data. Biological replicate structure: Replicates a and b were used in the regression analysis. 4 further available replicates (for GM12864, GM12865, GM12872, and GM12873) were used to add power for the allele-specific occupancy analysis, but were not used elsewhere. * Replicate “b” of GM12870 was only used for the linkage analysis.
(TXT)

Table S2 Pearson correlation of two replicates per individual of ChIP-seq data. Correlation performed after normalization at signal at ChIP-seq binding peaks.
(TXT)

Table S3 Summary of resequencing data mapped using bwa. Tags used in analysis indicates the uniquely aligned tags remaining after removing duplicates. On-target percent indicates the fraction of tags mapping back to within ±100 bp of a resequencing target.
(TXT)

Table S4 Survey of the effect of SNPs at transcription factor binding sites - 36,834 CTCF sites. SNPs are broken down by location relative to motif in the first two columns, either within the region of protein-DNA contact or outside it. The third column summarizes SNPs regardless of location relative to the motif; sites with multiple overlapping SNPs are counted once.
(XLS)

Table S5 Location of ChIP-seq binding positions in CEPH Pedigree 1459 called by SPP.
(TXT)

Table S6 Resequencing SNP calls in CEPH Pedigree 1459. chrom, chromStart, chromEnd ID, Strand, the 0-indexed hg18 location of the SNP. numNonRefAllelesGrandparents, number of nonreference alleles in 4 grandparents; genotype, nonreference / reference allele; GM12864-74, called genotypes with 00 indicating missing data.
(TXT)

Table S7 Sites tested in association analysis. chrom, chromStart, chromEnd, Strand, the strand-oriented hg18 coordinates of 134 bp window around the motif. peakID refers to the ChIP-seq peak; flag, nonzero indicates that the SNP was excluded from the analysis; position location of the SNP relative to motif; positionFlag, TRUE indicates position can not be reliably determined. allele.1, allele.2, and chimpAllele, the stronger, weaker and chimp alleles of the SNP. Slope, y.intercept, q.value refer to the regression. Signal.het, ChIP-seq density of the heterozygote class from regression model.
(TXT)

Table S8 Sanger validation of SNPs from capture resequencing. 33 sites tested exhibit no discordance between capture resequencing and Sanger validation. chrom, chromStart, chromEnd, Strand, the strand-oriented hg18 coordinates of the SNP; SNP ID, from Table S6; nonref/ref, alleles of the SNP; amplicon_start, amplicon_end, coordinates of predicted amplicon; Left PCR Primer, Right PCR Primer, primer sequences for PCR; genotypes of 12 samples, 00 indicates that Sanger sequencing failed; num_concordant, num_discordant, number of concordant and discordant genotypes for this SNP.
(TXT)

Table S9 Validation of SNPs from capture resequencing using HapMap genotypes. 4,246 sites tested exhibit 244 discordant calls (out of 27,808, 0.88%) between capture resequencing and public HapMap genotypes. chrom, chromStart, chromEnd, Strand, the strand-oriented hg18 coordinates of the SNP; SNP ID, from Table S6; nonref/ref, alleles of the SNP; genotypes of 6 samples, 00 indicates no HapMap data available; num_concordant, num_discordant, number of concordant and discordant genotypes for this SNP.
(TXT)
